# A Web-Based Intervention for Users of Amphetamine-Type Stimulants: 3-Month Outcomes of a Randomized Controlled Trial

**DOI:** 10.2196/mental.3278

**Published:** 2014-09-11

**Authors:** Robert J Tait, Rebecca McKetin, Frances Kay-Lambkin, Bradley Carron-Arthur, Anthony Bennett, Kylie Bennett, Helen Christensen, Kathleen M Griffiths

**Affiliations:** ^1^ National Drug Research Institute Faculty of Health Sciences Curtin University Perth Australia; ^2^ National Institute for Mental Health Research The Australian National University Canberra Australia; ^3^ Centre for Research on Ageing, Health and Wellbeing The Australian National University Canberra Australia; ^4^ National Drug and Alcohol Research Centre University of New South Wales Sydney Australia; ^5^ Centre for Translational Neuroscience and Mental Health University of Newcastle Newcastle Australia; ^6^ Black Dog Institute University of New South Wales and Prince of Wales Hospital Sydney Australia

**Keywords:** amphetamine related disorders, Internet, World Wide Web, randomized control trial, cognitive therapy, online, Web-based, motivational enhancement, intervention

## Abstract

**Background:**

Among illicit drugs, the prevalence of amphetamine-type stimulant (ATS) use is second only to cannabis. Currently, there are no approved pharmacotherapies for ATS problems, but some face-to-face psychotherapies are effective. Web-based interventions have proven to be effective for some substance use problems, but none has specifically targeted ATS users.

**Objective:**

The objective of the study was to evaluate the effectiveness of a Web-based intervention for ATS problems on a free-to-access site compared with a waitlist control group.

**Methods:**

We used a randomized controlled trial design. The primary outcome measure was self-reported ATS use in the past three months assessed using the Alcohol, Smoking, Substance Involvement Screening Test (ASSIST). Other measures included quality of life (EUROHIS score), psychological distress (K-10 score), days out of role, poly-drug use, general help-seeking intentions, actual help-seeking, and “readiness to change”. The intervention consisted of three fully automated, self-guided modules based on cognitive behavioral therapy and motivation enhancement. The analysis was an intention-to-treat analysis using generalized estimating equation models, with a group by time interaction as the critical assessment.

**Results:**

We randomized 160 people (intervention n=81, control n=79). At three months, 35/81 (43%) intervention and 45/79 (57%) control participants provided follow-up data. In the intervention group, 51/81 (63%) completed at least one module. The only significant group by time interaction was for days out of role. The pre/post change effect sizes showed small changes (range *d*=0.14 to 0.40) favoring the intervention group for poly-drug use, distress, actual help-seeking, and days out of role. In contrast, the control group was favored by reductions in ATS use, improvements in quality of life, and increases in help-seeking intentions (range *d*=0.09 to 0.16).

**Conclusions:**

This Web-based intervention for ATS use produced few significant changes in outcome measures. There were moderate, but nonsignificant reductions in poly-drug use, distress, days partially out of role, and increases in help-seeking. However, high levels of participant attrition, plus low levels of engagement with the modules, preclude firm conclusions being drawn on the efficacy of the intervention and emphasize the problems of engaging this group of clients in a fully automated program.

**Trial Registration:**

Australian and New Zealand Clinical Trials Registry: ACTRN 12611000947909; https://www.anzctr.org.au/Trial/Registration/TrialReview.aspx?ACTRN=12611000947909 (Archived by WebCite at http://www.webcitation.org/6SHTxEnzP).

## Introduction

### Global Assessment of Amphetamine Type Stimulant

Global assessments of illicit drugs place the prevalence of amphetamine type stimulant (ATS) use second only to cannabis, with an estimated 0.6% of the adult population thought to have used ATS in the last year [1]. In 2010, about 2.2% of Australian adults used methamphetamine/amphetamines and 3.1% used “ecstasy” in the last year, which are the main drugs encompassed by ATS [2]. This use translates into ATS being listed as the primary drug of abuse for more than 20% of those in treatment in Asia, 12% in North America, and 9% in Europe [3]. Even though the consumption of more potent types of ATS, such as crystalline methamphetamine, and utilization of more rapidly absorbed routes of administration (ie, smoking, injecting) have a high potential for developing dependence [4], most users do not reach diagnostic criteria. Therefore, interventions are needed across the spectrum from harm reduction for irregular “recreational” use through to treatment of stimulant use disorders [5].

Although ATS use is widespread, there is currently a lack of cost-effective scalable interventions that can be used to address dependence and other harms from ATS use [6], and no pharmacotherapy has yet been approved as a treatment of ATS dependence [7]. Currently, the treatment of ATS disorders relies on psychosocial interventions, with positive outcomes reported for the intensive application of psychological interventions such as contingency management, cognitive behavior therapy (CBT) and motivational interviewing (MI) [8]. Behavioral interventions can be extremely resource intensive; with some interventions requiring 156 weeks of treatment [8], so there have been attempts to develop shorter programs. Brief CBT based interventions, requiring up to four sessions, have resulted in significant reductions in amphetamine use and greater likelihood of abstinence than in control participants who just received a self-help booklet [9]. In Australia, it is estimated that 33% of dependent ATS users receive treatment for their ATS use in any year [10], and the high prevalence of lifetime comorbidity means that ATS users access health services more than those with other substance use disorders or other mental health disorders [11]. Nevertheless, traditional behavioral treatment options are not generally accessed by ATS users, who frequently report their needs are not being met in these settings [5].

### Evidence Base for eHealth Interventions

In the light of evidence that psychological interventions can reduce the use of ATS [8,9,12], there is potential to develop Web-delivered, mobile telephone or computer-based (henceforth referred to generically as “eHealth”) treatments for ATS users, an approach that has been effective with other conditions. The evidence base for the effectiveness of eHealth interventions for illicit drug use is limited, and we are not aware of any eHealth treatment interventions that currently exist specifically for ATS users. A review of interventions for cannabis use found only 10 studies that reported outcomes on cannabis consumption with an overall effect size of *g*=0.16 [13]. A review of eHealth interventions for drug use more broadly identified programs developed for specific drugs (eg, opiates) or interventions covering a range of illicit drugs including amphetamine, cannabis, cocaine, hallucinogens, inhalants, and opiates [14]. However, this review did not synthesize an overall outcome for their effectiveness, but concluded that there was evidence for their initial efficacy compared with control conditions [14].

Although not specifically ATS, one intervention has been evaluated among consumers of cocaine [15]. Having recruited 196 participants, the percentage who completed follow-up at 4, 6, and 26 weeks was 17%, 15%, and 6%, illustrating the difficulty of retaining this population in fully automated interventions [15]. Unsurprisingly, given the small sample retained (n=11 at 26 weeks) in the eHealth study, there were no significant time by group interactions on the key outcome measures, severity of dependence and craving [15]. Web-based or computer-based interventions can also be delivered as an adjunct to in-person treatment, which may serve to improve retention. Data from a mixed cohort of substance dependent individuals that received computer delivered (eg, at a clinic) CBT in addition to in-person treatment retained 72% at three months and 65% of participants at six months [16]. By comparison, a review of in-person interventions reported retention at three months ranging from 37% to 90% [6].

The aim of the current study was to evaluate a fully automated, self-guided Web-delivered intervention, derived from established psychological approaches (ie, CBT, MI), to reduce the use of ATS and associated problems at three months post intervention.

## Methods

### Design

We used a two-group randomized controlled trial, with the intervention group receiving a Web-delivered intervention comprised of three modules, which are described below. The wait-list control group received the same assessment procedures as the intervention group, but they were only able to access the intervention resources after six months. We also provided all participants with contact details for emergency services, such as Lifeline Australia. The full methodology has been described previously in detail [17].

### Sample

**Figure 1:**
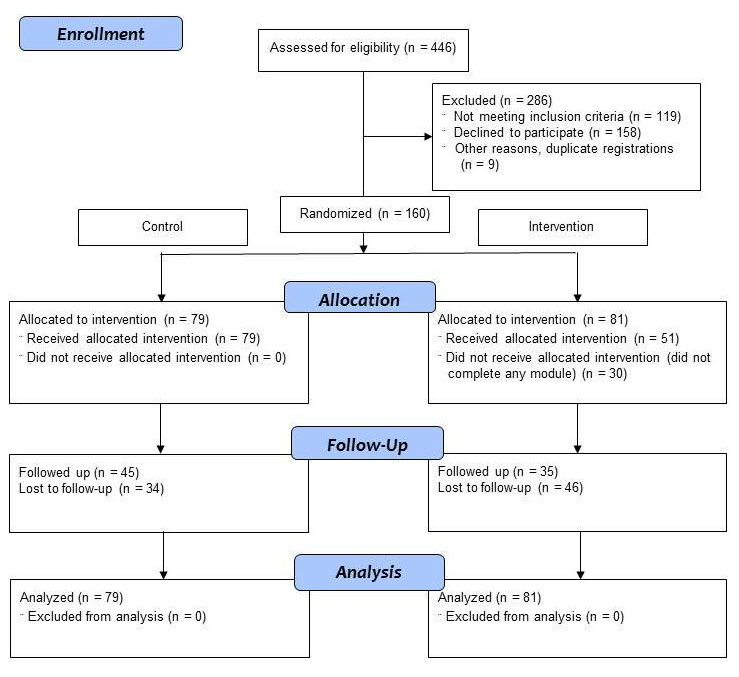
CONSORT flow diagram.

We recruited participants by advertising on social networking sites and posters in local clinics. To be eligible, participants had to be a resident of Australia, age 18 years or older, and to report use of ATS (eg, meth/amphetamine, ecstasy, nonmedical use of prescription stimulants) in the last three months. Given the nature of the intervention, participants required access to the Internet and a valid email address. We excluded those who were currently receiving any treatment for stimulant abuse/dependence or pharmacotherapy such as methadone, naltrexone, or buprenorphine for a substance use disorder (nicotine replacement therapy was permitted), or who reported a lifetime diagnosis of schizophrenia, schizoaffective, or bipolar disorder. In addition, nine cases were excluded as duplicate registrations (eg, duplicate Internet protocol addresses/email addresses/payment addresses. Inspection of log files by AB also indicated that these were likely to be repeated registrations). Figure 1 shows the flow of participants through the study. Recruitment opened in January 2013 and closed in July 2013. Of the 446 people assessed, 160 of 446 (35.8%) fulfilled the study criteria.

### Procedure

All stages of enrollment and screening were performed via the free study website. Eligible participants provided active consent by “clicking” on a box for each element of the consent form (see Multimedia Appendix 1). A personalized link to access the study was sent to verify their email address and to allow them to create a username and password. Participants were directed to a Web baseline survey before being randomized. We used a simple randomization process that was fully automated with permuted blocks of four with a one to one allocation ratio. Participants who were not eligible for the study were provided with information about other potentially useful websites and resources.

Those in the intervention group were given immediate access to the first module. Participants were advised to allow one week between modules, but could progress at their own pace, although each page in a module had to be opened in sequence to complete the module and obtain access to the next one. Reminder emails were sent three days after the scheduled start date if it had not been commenced, and at day seven when the next module was due. This was repeated for the third module. An email invitation to complete the follow-up assessment was sent after three months. Participants received AU$20 for baseline and follow-up assessments. The study received approval from The Australian National University Human Research Ethics committee and was registered with the Australian and New Zealand Clinical Trials Registry ACTRN 12611000947909.

### Modules

In developing the intervention, we drew on motivational interviewing and CBT methods that had been used in clinical practice with amphetamine users [18]. The approach was one of harm minimization, with participants able to decide on the most appropriate goals for themselves, for example, quitting completely, reducing their drug use, and using in a less hazardous manner. Module one explores the typical problems which ATS users incur, including: (1) relationships with family and friends, (2) health, (3) finances, (4) work/study, (5) legal issues, (6) mental health, and (7) specific drug use problems. The last page provides a summary of the endorsed problems, and guides the participant to generate a “map” of the interconnections between these issues. The second module requires participants to think about the pros and cons of their stimulant use, and the likely good and bad things related to changing their behavior, and draws on the Miller and Rollnick model [19]. To aid in their “decision balance” for each element that they select, participants rate its importance. The last module focuses on behavioral change, including techniques such as: (1) setting clearly specified goals, (2) actions on specific dates, (3) strategies to help with controlling and overcoming cravings, (4) refusal skills, (5) managing a “slip”, and (6) an action plan to deal with high risk situations. Sample images from the intervention are available elsewhere [17] and in Multimedia Appendices 2-5.

### Measures

All the study measures were self-report. The primary outcome measure was ATS use evaluated with the Alcohol, Smoking, Substance Involvement Screening Test (ASSIST) [20]. The ASSIST assesses lifetime and last three month use of nine drug categories (ie, tobacco, alcohol, cannabis, cocaine, ATS, inhalants, sedatives, hallucinogens, opioids, other). Data include frequency of use, cravings, problems (health, social, legal, or financial), failure to fulfill roles, concern expressed about their drug use, and if the person has ever tried and failed to control their drug use. Finally, injection of drugs was assessed. The standard ASSIST scoring algorithm was used to calculate a score for ATS use (range 0-39) [20].

We assessed secondary outcomes in terms of: (1) help-seeking intentions (general help-seeking questionnaire, GHSQ) [21]; (2) actual help-seeking questionnaire (AHSQ) [22,23]; (3) readiness to change, modified to assess ATS rather than alcohol (Readiness to Change Questionnaire, RTCQ) [24]; (4) psychological distress (Kessler-10 questionnaire, K-10) [25]; (5) poly-drug use measured by the ASSIST [20]; (6) days out of role [26]; and (7) quality of life (European Health Interview Survey, EUROHIS, Quality of Life scale) [27]. We also collected demographic information (eg, age, sex, marital status), drug use history (eg, age of first use of ATS), and severity of dependence (Severity of Dependence Scale, SDS) [28].

The RTCQ has four items relating to each of the stages, “precontemplation”; “contemplation”; and “action”. The five point scales were summed to obtain scores for each stage, with participants designated to their highest scoring stage, or in the event of tied scores, the higher stage [24]. Psychological distress was indexed as the total score (range 10-50) on the K-10 [25]. Poly-drug use was the sum of ASSIST classes of drugs endorsed, excluding ATS use [20]. The GHSQ asked, “How likely is it that you would seek help from each of the following people for any amphetamine or other drug use problems during the next 4 weeks?”, and provided a list of nine potential sources of help (eg, friend, mental health professional, other). The seven point scale ranged from extremely unlikely (1) to extremely likely (7). The AHSQ asked, “Which of the following people have you gone to for advice or help in the past 2 weeks for any amphetamine or other drug use problems?”, and listed the same nine sources as the GHSQ. “Days out of role” was based on Kessler’s questions, but referencing “ATS drug use (eg, methamphetamine, ecstasy, ice)” rather than “depression” [26], and quality of life was the total EUROHIS score [27].

### Sample Size

The study was designed to detect a medium effect (eg, *d*=0.5) [29] with power of 0.8, which requires a sample of 60 people per group; to allow for 20% attrition, we recruited 80 people per group. In estimating the sample size, we drew on findings for stimulant users who were recruited in primary care settings and received a brief intervention in the ASSIST development study [20]. That group may be less heterogeneous than the current sample.

### Analysis

The primary analysis was an intention-to-treat (ITT) analysis and used generalized estimating equation (GEE) models. This approach overcomes many of the limitations of standard repeated measures analysis of variance. It uses all available data without requiring substitution or estimation of missing independent variables to avoid the exclusion of cases with noncomplete data and does not assume homogeneity of correlations over waves of measurement [30,31]. For continuous data, an unstructured correlation matrix was used together with a normal distribution and identity link. Categorical outcome measures were evaluated using GEE models with a multinomial distribution and cumulative logit link. After inspection of the data, days out of role, intended help-seeking, and number of people actually sought help from, were assessed using a Poisson distribution with a log link due to the positively skewed distribution. Outcomes were tested as the group (intervention, control) by time (baseline, three months) interaction. Due to significant differences in baseline data (see Table 1), actual help-seeking was included as a covariate, along with SDS, due to its importance in predicting attrition (see “Follow-Up” section). The primary outcome measure was the ASSIST ATS score, with other measures deemed as secondary outcomes.

A sensitivity analysis was conducted using multiple imputation of missing data using fully conditional specification with an iterative Markov chain Monte Carlo method. Maximum and minimum values were logically constrained, for example, to the possible range of scores on questionnaires. Baseline outcomes, plus demographic variables were used as predictors; three month outcomes were dependent and predictor variables in generating the 25 datasets. Effect sizes were calculated as the difference in pretest posttest means for the two conditions, divided by their common pretest standard deviation, multiplied by a bias correction factor (1-(3/4(N _treatment_+ N _control_-2)-1), Monte Carlo modeling shows that this provides the best estimate of the population effect from the commonly used effect size measures [32]. In addition, attrition was modeled with logistic regression to investigate the characteristics of those lost to follow-up. Baseline predictors were study group, RTC group, age, age of first ATS use, gender, SDS, K-10, ASSIST ATS, poly-drug use scores, actual help-seeking scores, and intended help-seeking scores. Finally a “per protocol” analysis was conducted to evaluate the effect of completing at least one module of the intervention. The ITT and imputed analyses were conducted blind to study condition by RJT.

## Results

### Group Characteristics

The characteristics of the two groups and overall sample at baseline are shown in Table 1. On all measures, the two groups reported similar baseline scores, except actual help-seeking, where the control had significantly higher levels. There were (38/60) 23.8% of the participants that were female; the mean age was 22.4 (SD 6.3). About 1/3 of users only consumed ATS occasionally (1-2 times in the last three months), with (62/160) 38.8% using ATS weekly or more frequently. Based on a SDS threshold score of five or more, (57/160) 35.6% participants were classified as “dependent”. The large majority had never injected drugs (137/160, 85.6%).

**Table 1:** Baseline characteristics by study group and sample.

Variable		Control n=79, n (%) or mean (SD)	Intervention n=81, n (%) or mean (SD)	Total N=160, n (%) or mean (SD)	Statistic
**Sex**					
	Female	21 (27)^a^	17 (21)	38 (24)^a^	χ^2^_1_=1.80; *P*=.41
Age		22.5 (7.1)	22.2 (5.5)	22.4 (6.3)	*t*_158_=0.34; *P*=.74
**Education**					
	Primary	2 (3)	6 (8)	8 (5)	χ^2^_3_=3.57; *P*=.31
	Secondary	50 (67)	50 (63)	100 (65)	
	Trade/technical	13 (17)	9 (11)	22 (14)	
	University	10 (13)	15 (19)	25 (16)	
**Employment**					
	Full-time	13 (17)	15 (19)	28 (18)	χ^2^_3_=0.60; *P*=.90
	Part-time	17 (22)	14 (18)	31 (20)	
	Unemployed	17 (22)	16 (21)	33 (21)	
	Student	30 (39)	33 (42)	39 (41)	
**Amphetamine Type Stimulants (ATS) frequency last 3 months**					
	1-2	27 (34)	20 (25)	47 (29)	χ^2^_3_=6.41; *P*=.09
	Monthly	18 (23)	33 (41)	51 (32)	
	Weekly	23 (29)	21 (26)	44 (28)	
	Daily/almost daily	11 (14)	7 (9)	18 (11)	
Age 1st ATS use		18.6 (4.2)	17.7 (2.6)	18.1 (3.5)	*t*_158_=0.08; *P*=.93
ATS score		16.8 (11.1)	17.0 (10.1)	16.9 (10.6)	*t*_158_=1.72; *P*=.09
Poly-drug use		4.6 (1.6)	4.8 (1.8)	4.7 (1.7)	*t*_158_=0.95; *P*=.34
Intended help-seeking		20.4 (10.9)	19.7 (11.2)	20.1 (11.0)	*t*_158_=0.40; *P*=.69
Actual help-seeking		0.8 (1.3)	0.3 (0.7)	0.6 (1.1)	*t*_113_=2.83 ^b^; *P*=.01
Kessler-10 (K-10) score		22.3 (8.3)	22.2 (8.4)	22.2 (8.3)	*t*_158_=0.02; *P*=.98
**Injected any drug**					
	Never	69 (87)	68 (84)	137 (86)	χ^2^_2_=0.58; *P*=.75
	Yes, not last 3 months	4 (5)	4 (5)	8 (5)	
	Yes, last 3 months	6 (8)	9 (11)	15 (9)	
Days out of role		2.9 (5.9)	3.5 (5.6)	3.2 (5.7)	*t*_158_=0.63; *P*=.53
Days part out of role		3.2 (4.8)	3.9 (5.3)	3.6 (5.3)	*t*_158_=0.79; *P*=.43
Quality of life		28.2 (5.8)	27.2 (6.3)	27.7 (6.1)	*t*_158_=0.99; *P*=.32
**Readiness to Change Questionnaire (RTCQ)**					
	Precontemplation	32 (41)	27 (33)	59 (37)	χ^2^_2_=2.83; *P*=.24
	Contemplation	24 (30)	35 (43)	59 (37)	
	Action	23 (29)	19 (24)	42 (26)	
Severity of Dependence (SDS)		3.8 (3.3)	3.7 (3.5)	3.7 (3.4)	*t*_158_=0.17; *P*=.86
SDS >5		33 (42)	24 (30)	57 (36)	χ^2^_1_=2.57; *P*=.11

^a^One person reported sex as “other”

^b^Levene’s correction for inequality of variances

^b^Missing data, education n=5, employment n=5

### Engagement

From the 81 intervention participants, 51/81 (63%) completed, 13/81 (16%) started, and 17/81 (21%) did not attempt the first module. The second module was completed by 45/81 (56%) participants and started by another 2/81 (2%); the respective figures for the third module were 39/81 (48%) and 4/81 (5%). Thus, 39/81 (48%) completed all the modules, 6/81 (7%) completed two modules, and six completed one module.

### Follow-Up

At three months, 45/79 (57%) participants from the control and 35/81 (43%) from the intervention completed follow-up surveys (Figure 1) (χ^2^_1_=3.03 *P*=.08). The proportion who submitted follow-up data in the intervention group varied with the number of modules completed, 7 (23%) who completed no modules, 2 (33%) who completed one module, 4 (67%) who completed two modules, and 22 (56%) who completed all three, Fisher’s exact test 9.21, *P*=.02. Logistic regression showed that “loss to follow-up” was not significantly related to group allocation. However, higher depression scores increased the odds of completing follow-up (odds ratio, OR) 1.06, 95% CI 1.00-1.11), while the odds were reduced with higher baseline poly-drug use OR 0.75 (95% CI 0.60-0.93), or higher baseline SDS OR 0.82 (95% CI 0.67-0.99).

### Intention-to-Treat Analyses

The results of the ITT analyses showed that there was only one significant group by time interaction for the outcome measures (Table 2). Those in the intervention group had a reduction in days out of role relative to the control group (estimated marginal mean, EMM, baseline 3.3, standard error, SE, 1.6; three months 0.70, SE 0.43; vs control EMM 3.1, SE 1.6; 2.9, SE 2.0). Table 2 also shows the scores on the outcome measures at three months together with the effect sizes. To facilitate the interpretation of the effect sizes, the group favored by the change is noted in the Table, because “improvements” constitute increases on some measures (eg, EUROHIS) and decreases on others (eg, ATS use). The majority of effect sizes favored the intervention group. We observed that actual help-seeking was lower for the intervention group at both time points, but their mean level increased while the mean decreased for the controls. With respect to RTC category, the proportion in the “precontemplation” stage fell in the intervention group (27/81, 33% to 8/34, 24%) and remained stable in the control group (32/79, 41% to 19/45, 42%). Changes in the proportions in the “action” and “contemplation” stages were similar for the two groups. The results for the pooled data after multiple imputations showed similar outcomes to the main analyses with only one significant group by time interaction. The intervention group showed improved outcomes relative to the control group for actual help-seeking (*P*=.02; intervention EMM baseline 0.32, SE.09; three months 0.84, SE 0.22; vs control EMM 0.74, SE 0.20; 0.87, SE 0.23.

**Table 2:** OR for group by time interaction plus posttest outcomes and pre/posttest effect sizes. Group by time interaction is adjusted for the SDS score at baseline and time varying actual help-seeking. Effect size is the difference in pre/post means/common pretest SD with bias correction factor [32]

Variable		OR (95% CI) group * time, mean (SD) or n (%)	Control n=45, mean (SD) or n (%)	Intervention n=35, mean (SD) or n (%)	Effect *d*	*P* value	Group favored
ATS score		0.70 (0.02, 24.64)	13.5 (10.0)	15.3 (9.3)	-0.16	.84	Control
Poly-drug use		0.51 (0.24, 1.09)	4.6 (1.7)	4.2 (1.8)	0.40	.08	Intervention
Intended help-seek		0.91 (0.72, 1.16)	19.5 (9.1)	18.1 (7.3)	0.09	.46	Control
Actual help-seek		1.90 (0.82, 4.39)	0.69 (.95)	0.57 (.92)	-0.33	.14	Intervention
K-10 score		0.15 (0.01, 1.97)	21.6 (7.7)	20.3 (7.4)	0.15	.15	Intervention
Days out of role		0.22 (0.07, 0.68)	2.2 (5.1)	1.1 (2.1)	0.29	.01	Intervention
Days part out of role		0.45 (0.15, 1.33)	3.0 (5.5)	2.9 (6.1)	0.14	.15	Intervention
Quality of life		1.99 (0.31, 12.82)	29.8 (5.4)	28.2 (5.0)	0.11	.47	Control
**RTCQ**							
	Precontemplation	0.55 (0.21, 1.42)	19 (42)	8 (24)	-	.22	Intervention
	Contemplation	-	7 (16)	9 (27)			
	Action	-	19 (42)	17 (50)			

### “Per Protocol” Analysis

The final analysis compared those who completed one or more modules (n=28), zero modules (n=7), or who were in the control group (n=45). Of the outcome measures, only actual help-seeking showed a significant interaction effect (OR 2.90, 95% CI 1.10-7.62). Actual help-seeking increased in those who undertook at least one module (baseline mean 0.22, SE 0.08; three months 0.59, SE 0.21), whereas for both the control group (baseline 0.72, SE 0.22; three months 0.67, SE 0.25) and the zero module group (baseline 0.42, SE 0.15; three months 0.14, SE 0.15) actual help-seeking decreased. Note, analysis of “per protocol” data does not represent randomized outcomes.

## Discussion

### Principal Results

To the best of our knowledge, this is the first Web-based intervention developed specifically for users of ATS. There was only one (time by group) significant change on any of the key outcome measures, with an improvement in the number of days out of role for the intervention group. Further, the effect sizes for the intervention were smaller than those estimated in the design phase. The findings of the multiple imputations analysis reinforce the conclusion that the study was “insufficiently powered” to detect small effects. Nevertheless, to put these outcomes into perspective, the effects were larger than those recently reported for eHealth interventions for cannabis use [13] and similar to those for brief face-to-face interventions for alcohol use problems [33]. Thus, there is the potential that this intervention could be of benefit to users of ATS, at least among those with similar characteristics to this cohort. Nevertheless, the high level of attrition and low level of engagement limit the conclusions that can be drawn from these data. Improving engagement is a critical goal for interventions with substance using groups.

Moving beyond the ITT analysis, the effect size analysis and the per-protocol analysis (eg, those completing one or more modules) suggest that the intervention increases actual help-seeking behavior in participants. A recent review has found that it is difficult to change help-seeking, even where this is a specific aim of the intervention, at least among samples with common mental health disorders (eg, depression, anxiety) [34]. Brief interventions can result in small increases in help-seeking in those with alcohol use problems, but are more effective in those without comorbid mental health disorders [35]. This reinforces the above review, which found effect sizes ranging from *d* =−.02 to .24 for changing help-seeking [34]. Therefore, the significant effects found in the per-protocol and multiple imputed data analyses of the current study are an important outcome for a low intensity intervention. Further research is required to evaluate if this type of Web-based intervention can be effectively integrated into a stepped-care program for ATS users as previously recommended [5].

Given that the intervention specifically targeted and was designed for users of ATS, it is surprising that the control group had a greater decline in ATS use than the intervention group, especially as the latter showed a greater reduction than the control in poly-drug use, as indexed by the number of different categories of drugs used in the last three months (excluding ATS). The mean number of drug types used by participants was four to five in addition to ATS. That the participants appear to be opting to change other drug use, as shown by reduced poly-drug scores, rather than their ATS use is of concern, as even low frequency of exposure (ie, greater than five) to ATS is associated with the development of stimulant use disorders [11].

### Limitations

There are a number of limitations that need to be acknowledged in the interpretation of these findings. The sample would be regarded as having less severe substance use problems, with the large majority having never injected any drug and their severity of dependence scores being low compared with ATS treatment seeking groups (eg, 75% injecting ATS, mean severity of dependence score approximately 9.0) [36]. Thus, care should be taken in extrapolating beyond this type of ATS user. Nevertheless, 57 participants scored five or more on the SDS and, on the basis of this screening measure, are likely to be ATS dependent [37]. The loss to follow-up of a significant proportion of participants threatens the internal validity of the study. Although this was not related to group allocation in a logistic model, the association with increased severity of dependence and poly-drug use reinforces the caveat that this type of low intensity intervention may not be suitable for those with more severe drug use problems, consistent with the broader literature on brief interventions for substance use [33,38]. Indeed, the small effect sizes reported for eHealth interventions with cannabis users [13] could imply that more intensive interventions are required for most illicit drug users. Other eHealth interventions with illicit drug users (cocaine) have encountered more extensive attrition [15], but the results obtained in the current study are comparable with in-person interventions for ATS [6] and consistent with the broader literature from fully automated Internet interventions [39]. Differences between the groups in the proportion followed-up may also threaten the internal validity.

A further concern is the low level of engagement with the intervention (30/81) 37% of participants randomized to the intervention did not complete the first module, and future research is required to investigate ways to encourage intervention completion. Although in a radically different sample (adolescent girls), recruiting mother-daughter dyads has achieved remarkable retention rates [13]; recruiting “user-significant other” dyads might improve retention in other drug use groups. This is particularly important given previous findings that completion of at least one in-person module of a four-session intervention for ATS was associated with greater ATS reductions than those who did not return for any sessions [9], in addition to our finding here that actual help-seeking increased for people completing at least one module of the Internet intervention. Similar findings have also been reported for Internet support interventions where completion of a greater number of modules following residential treatment was associated with better posttreatment outcomes [40]. Finally, the low level of engagement diminishes any potential difference between the study groups.

### Conclusions

The impact of eHealth treatment interventions for ATS drug use remains open to question due to the small effects associated with their application and their potential clinical relevance. However, the impact of an intervention relates both to the prevalence of the condition and its consequences. Thus, brief interventions by primary care physicians have a net benefit of only 1%-3% in the cessation of smoking, but are still cost effective and recommended [41,42]. The potential of eHealth interventions to reach those unable or unwilling to access conventional facilities means that they should be further evaluated in large scale trials, including effectiveness trials to determine if people will use them without research incentives. It also seems warranted to evaluate their effect as an adjunct to conventional treatment. Ways to further increase engagement with Internet-based treatment programs require research attention, particularly given the current debate as to whether or not “supported” or “guided” eHealth interventions (ie, involving some input from a therapist) are more effective than unguided programs [43,44]. Including an easy means of providing feedback at the end of each module could elicit data to modify the intervention and, hence, improve the experience for users. Without dramatic improvements in retention, substantially larger studies will be required to detect small differences between groups, but which will still leave results with questionable internal validity.

## Appendix

Multimedia Appendix 1 Online information and consent form.

Multimedia Appendix 2 Breakingtheice introduction page.

Multimedia Appendix 3 Module 1.

Multimedia Appendix 4 Module 2.

Multimedia Appendix 5 Module 3.

Multimedia Appendix 6 CONSORT-EHEALTH checklist V1.6.2 [45].

## Abbreviations

AHSQ: actual help-seeking questionnaire
ASSIST: Alcohol, Smoking, Substance Involvement Screening Test
ATS: amphetamine-type stimulants
CBT: cognitive behavior therapy
EMM: estimated marginal mean
EUROHIS: European Health Interview Survey
GEE: generalized estimating equation
GHSQ: general help-seeking questionnaire
ITT: intention-to-treat
K-10: Kessler-10 questionnaire
MI: motivational interviewing
NHMRC: National Health and Medical Research Council
RTCQ: Readiness to Change Questionnaire
SDS: Severity of Dependence Scale
SE: standard error
