# Supplementary material for: A Web-Based Intervention for Users of Amphetamine-Type Stimulants: 3-Month Outcomes of a Randomized Controlled Trial
Source: JMIR Ment Health. 2014 Sep 11;1(1):e1. doi: 10.2196/mental.3278 (PMC4607377; doi:10.2196/mental.3278)
Supplement: Multimedia Appendix 1 [file mental_v1i2e1_app1.pdf]

breakingtheice

An Internet-based Intervention To Reduce Stimulant Use

We are testing a web-based intervention for people who want to reduce their use of stimulants such as meth/amphetamines or ecstasy. The objective of the study is to test if this is helpful for people who use stimulants either occasionally or regularly.

Who can take part?

We are looking for people aged 18 or older, who have used any amphetamine type stimulant (e.g. meth/amphetamine, ecstasy or non-medical use of prescription stimulants) in the last three-months. You must be living in Australia.

What does the research involve?

First, we'll collect some basic information about your use of drugs and your mental health to make sure that the study is suitable for you. If it is, we'll ask for your consent to collect information about you and a contact e-mail address so that we can send you a personal user name and password. You will then be randomised to either an on-line intervention or control group where we'll ask you to do 3 surveys over the next 6 months.

If you are in the intervention group, we'll ask you to complete three modules that involve quizzes, asking about your goals and recommended changes that you may like to make to your lifestyle. Each module will take about 30 minutes to complete. After three and six months, we'll e-mail you again to find out how you are progressing, to ask what you thought of the web-site and about your use of stimulants.

Your participation in this study is completely voluntary – you are free to withdraw from this project at any time without giving a reason and without penalty.

Are the results confidential?

Every phase of the project is carried out in accordance with Australian National Health and Medical Research Council Guidelines. This project has been approved by the ANU Human Research Ethics Committee (Protocol 2011/358).

All the survey information you provide will be stored with an ID number only, and files linking your ID number with login name are stored separately. Any information you enter as part of the intervention will also be stored separately from your email address. Any information that is obtained and that can be identified with you will remain confidential and will be disclosed only with your permission or except as required by law.

Online survey answers and online intervention user data will be recorded and stored in a password protected computer at the Australian National University and are only accessible to authorised project staff. We are committed to maintaining the security of all the information that will be collected throughout this project. Any user of the Internet and the World Wide Web should be aware that it is an insecure public network that gives rise to a potential risk that a user's transactions are being viewed, intercepted or modified by another party or that data which the user downloads may contain computer viruses or other defects.

The results of the project may be published in academic journal articles. Only group results for survey data will be reported, no information that can identify any individual will be published.

Is there any benefit from taking part?

We hope that you will find your participation helpful. However, we'll give you $20 for completing each of the 3 surveys in appreciation for your time in answering our questions. In order to send you a gift voucher, we will need an e-mail address, or if you would prefer us to send a cheque we'll need a postal address. This will not be linked to your study information. You may choose to ensure that your e-mail address doesn't make you easy to identify – for example you may consider using a temporary 'hotmail' address if you are concerned.

Are there any risks?

There are no specific risks from taking part in this research, but if it raises emotional or drug related questions, or distresses you, there are community organisations that can provide help or advice

For drug problems

    www.reachout.com
    Alcohol and Drugs Information Service ACT 24 hour help line (02) 6207 9977

For personal distress

    Lifeline 13-11-14 www.lifeline.org.au

What if I have a question or concern about the project?

If you have any questions about this project please contact Dr Rebecca McKetin (02 61258407 or Rebecca.McKetin@anu.edu.au). If you have any concerns about the way the research was conducted please contact the Secretary, Human Research Ethics Committee, Research Office, Chancelry 10B, The Australian National University, ACT 0200 (02-6125-7945 or human.ethics.officer@anu.edu.au).

The senior researcher is Dr Robert Tait, Centre for Mental Health Research, in collaboration with the National Drug and Alcohol Research Centre. The Commonwealth Department of Health and Ageing funded this research.

ANU website's disclaimer and privacy statement

You can view the ANUs privacy statement at http://www.anu.edu.au/legal/content/copyright.html

In addition, our Centre has developed ways to protect the security of personal information collected during the course of on-line studies. The conduct of the Centre on personal information we collect is governed by the Privacy Act 1988. The Act lays down a set of Information Privacy Principles that oblige us to treat all the information given to us by participants with the utmost respect, and to take every reasonable measure to ensure that their confidence is never breached.

Consent

I have read the information page provided and agree to participate in the Breaking the Ice project. ☐ Yes

Before answering the questions on this page, I have been given the opportunity to ask questions related to any possible harm I might suffer as a result of my participation in the project. I have received satisfactory answers to any questions that I have asked. ☐ Yes

I understand that my participation is completely voluntary and that I am free to withdraw from the study at any time without prejudice. ☐ Yes

I understand that research data gathered from the results of the study may be published, provided that I cannot be identified. ☐ Yes

I understand that if I have concerns about the scientific aspects of the study, I can contact Dr Rebecca Mcketin on (02) 6125 8407, who will be happy to answer my concerns. ☐ Yes

I understand that I can print this consent form and Participant Information Sheet statement for future use. ☐ Yes

I understand that ethical concerns may be directed to: ☐ Yes
The Ethics Secretariat
Human Research Ethics Committee, Research Office, Chancellery 10B,
The Australian National University, ACT 0200
Phone: 6125 7945
Email: Human.Ethics.Officer@anu.edu.au [LINK to email]

I am aged 18 years or older. ☐ Yes

I am currently living in Australia. ☐ Yes
