# Supplementary figures and images for: A Web-Based Intervention for Users of Amphetamine-Type Stimulants: 3-Month Outcomes of a Randomized Controlled Trial
Source: JMIR Ment Health. 2014 Sep 11;1(1):e1. doi: 10.2196/mental.3278 (PMC4607377; doi:10.2196/mental.3278)

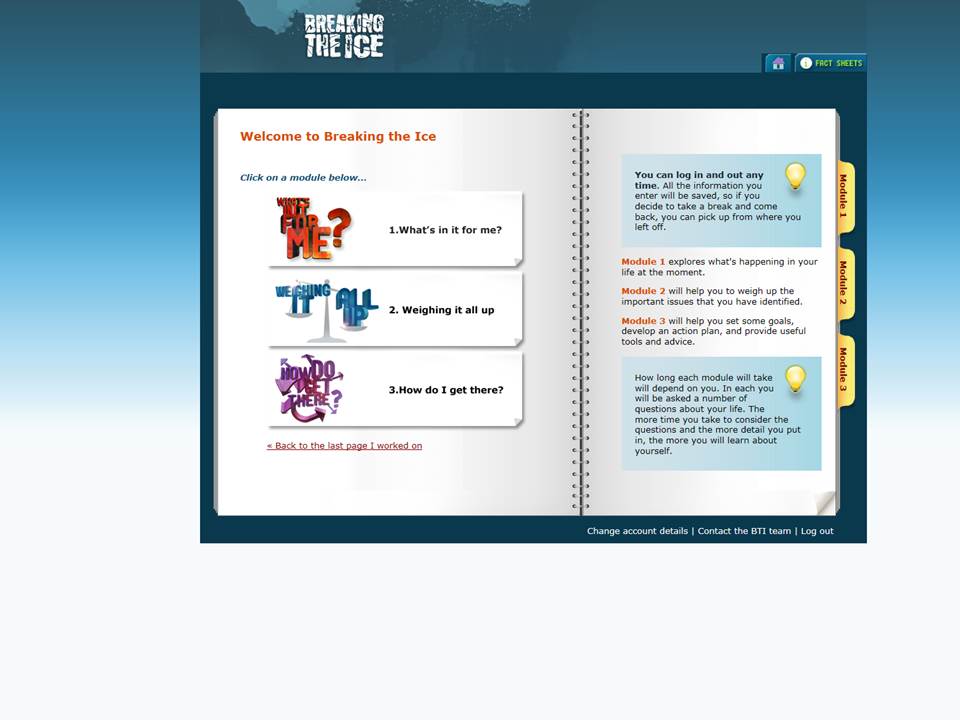

Supplement: Multimedia Appendix 2 [file mental_v1i2e1_app2.JPG]

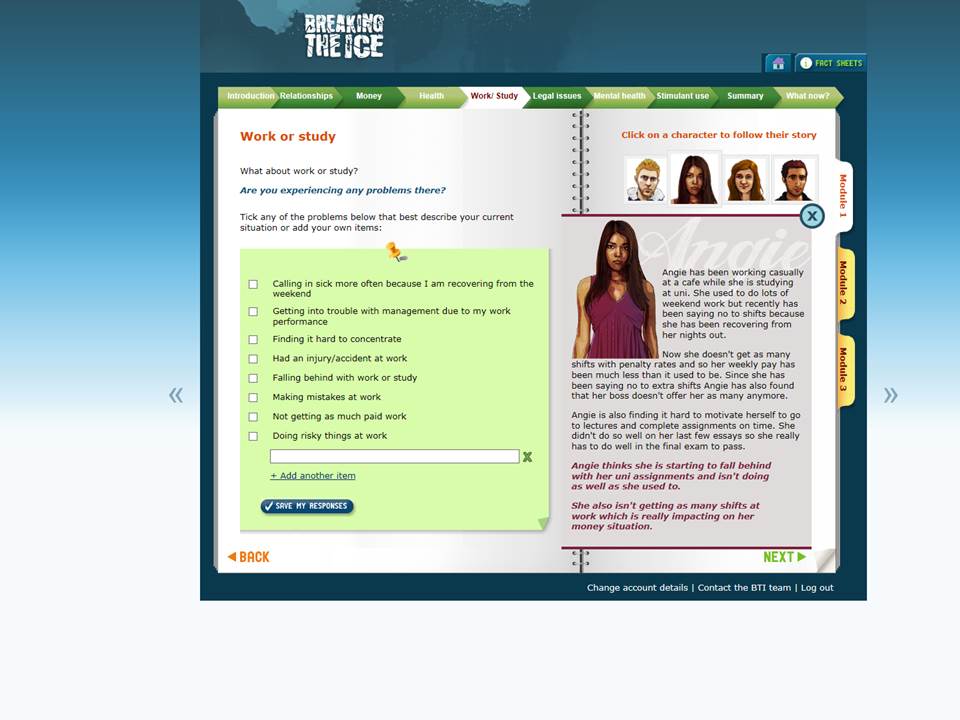

Supplement: Multimedia Appendix 3 [file mental_v1i2e1_app3.JPG]

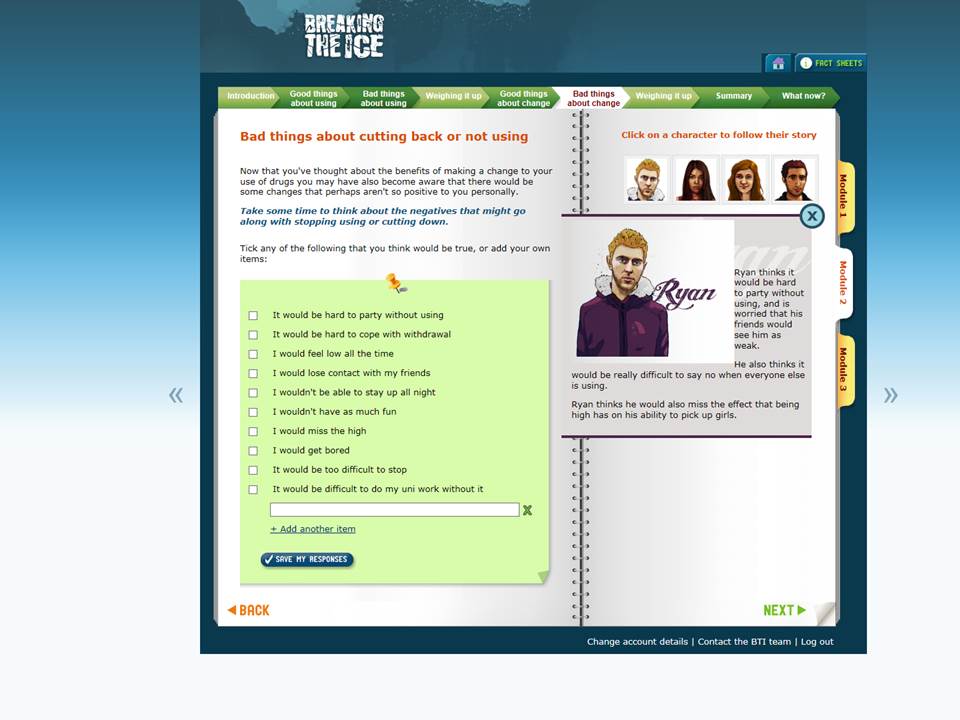

Supplement: Multimedia Appendix 4 [file mental_v1i2e1_app4.JPG]

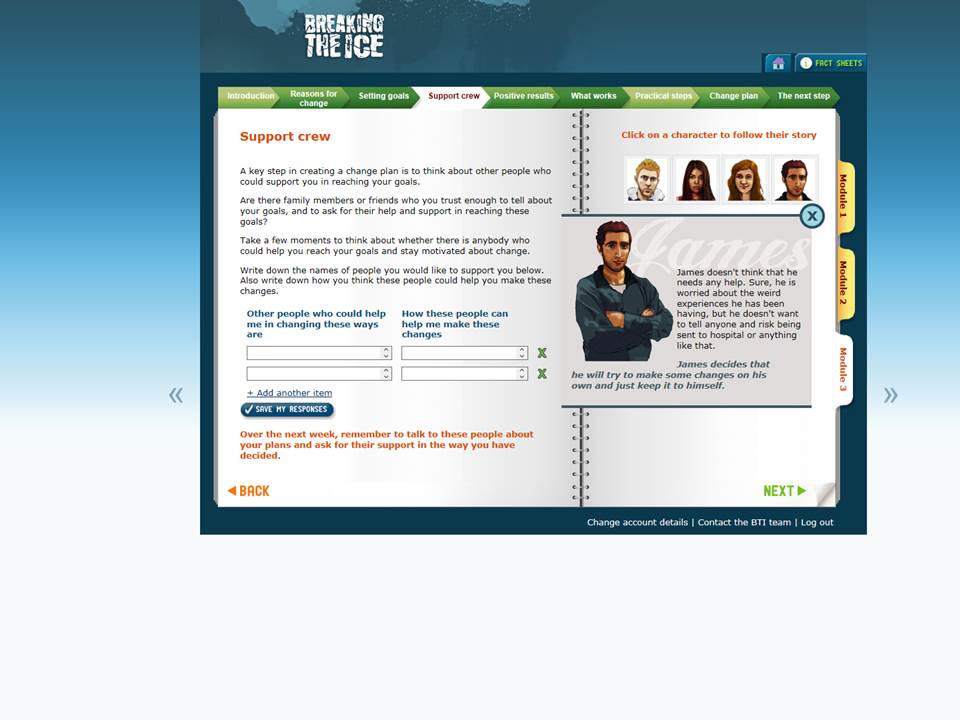

Supplement: Multimedia Appendix 5 [file mental_v1i2e1_app5.JPG]
