# Supplementary material for: A Web-Based Intervention for Users of Amphetamine-Type Stimulants: 3-Month Outcomes of a Randomized Controlled Trial
Source: JMIR Ment Health. 2014 Sep 11;1(1):e1. doi: 10.2196/mental.3278 (PMC4607377; doi:10.2196/mental.3278)
Supplement: Multimedia Appendix 6 [file mental_v1i2e1_app6.pdf]

CONSORT-EHEALTH Checklist V1.6.2 Report

(based on CONSORT-EHEALTH V1.6), available at [http://tinyurl.com/consort-ehealth-v1-6].

3278

Date completed

8/8/2014 5:05:59

by

Robert Tait

A randomised controlled trial of a web-based intervention for users of amphetamine-type stimulants: Six month outcomes (part II)

TITLE

1a-i) Identify the mode of delivery in the title

"A randomised controlled trial of a web-based intervention for users of amphetamine-type stimulants: Six month outcomes"

1a-ii) Non-web-based components or important co-interventions in title

n/a waitlist control used

1a-iii) Primary condition or target group in the title

"users of amphetamine-type stimulants"

ABSTRACT

1b-i) Key features/functionalities/components of the intervention and comparator in the METHODS section of the ABSTRACT

"effectiveness of a self-guided web-based intervention".....".

" comparing a waitlist control with a fully automated intervention containing three modules derived from cognitive behavioral therapy and motivation enhancement"

1b-ii) Level of human involvement in the METHODS section of the ABSTRACT

" self-guided"

1b-iii) Open vs. closed, web-based (self-assessment) vs. face-to-face assessments in the METHODS section of the ABSTRACT

"a free-to-access site"

1b-iv) RESULTS section in abstract must contain use data

"We randomized 160 people (intervention n=81, control n=79). At six months, 38 (47%) intervention and 41(52%) control participants provided data"

1b-v) CONCLUSIONS/DISCUSSION in abstract for negative trials

"This self-guided web-based intervention encouraged help-seeking associated with ATS use, but it did not reduce ATS use"

INTRODUCTION

2a-i) Problem and the type of system/solution

"In Australia it is estimated that 97000 people are dependent on ATS but few specialist treatment services are available [5]: data suggest that just 16% of non-dependent and about 30% of dependent methamphetamine users received any treatment for their drug use in the previous year [6, 7]. With no pharmacotherapy currently approved for ATS disorders, treatment relies on face-to-face interventions,...which can be extremely resource intensive, preventing their widespread implementation"

2a-ii) Scientific background, rationale: What is known about the (type of) system

"There has been considerable interest in the development and evaluation of web-based interventions for tobacco use or alcohol consumption, with a recent systematic review summarizing data from studies involving nearly 40000 smokers [16] and, a review of online interventions for alcohol finding 17 studies, comprising nearly 10000 participants [17]. However, the development of internet interventions for illicit drug use is at a more formative stage"

METHODS

3a) CONSORT: Description of trial design (such as parallel, factorial) including allocation ratio

"It was hypothesized that the intervention group would have a greater reduction in their use of ATS six months after starting the intervention than the control group. We also examined whether the intervention resulted in improvements on a range of secondary outcomes"

3b) CONSORT: Important changes to methods after trial commencement (such as eligibility criteria), with reasons

none

3b-i) Bug fixes, Downtimes, Content Changes

none

4a) CONSORT: Eligibility criteria for participants

"To be eligible, participants had to be resident in Australia, aged 18 years or older and report use of ATS (e.g. meth/amphetamine, ecstasy, non-medical use of prescription stimulants) in the previous three months. Due to the nature of the intervention, participants were required to have access to the internet and to provide a valid email address. We excluded potential participants if they reported that they were currently receiving any treatment for stimulant abuse/dependence or methadone, naltrexone or buprenorphine for a substance use disorder. Those who reported that a doctor had ever diagnosed them as having schizophrenia, schizoaffective, or bipolar disorder were also excluded. Finally, we inspected registration details and nine cases were excluded as duplicate registrations"

4a-i) Computer / Internet literacy

Not literacy per se but "Due to the nature of the intervention, participants were required to have access to the internet and to provide a valid email address"

4a-ii) Open vs. closed, web-based vs. face-to-face assessments:

"All participants were screened and enrolled via the free study website"

"Outcome data were collected at three and six months and were all based on self-report."

4a-iii) Information giving during recruitment

"Those who fulfilled the eligibility criteria were invited to provide consent by 'clicking' an onscreen box for each element of the consent form. After consent, the website generated a personalized link that was sent to the participant's email address. This enabled the participant to create their own study username and password. Next, participants were directed to an online baseline survey. Finally, they were randomized "

Consent and information sheet (accepted for publication JMIR Mental Health

4b) CONSORT: Settings and locations where the data were collected

"participants had to be resident in Australia"

4b-i) Report if outcomes were (self-)assessed through online questionnaires

""Outcome data were collected at three and six months and were all based on self-report.""

4b-ii) Report how institutional affiliations are displayed

Not addressed specifically - the institution is clearly identified in the information / consent form and the logo was on online advertising.

5) CONSORT: Describe the interventions for each group with sufficient details to allow replication, including how and when they were actually administered

5-i) Mention names, credential, affiliations of the developers, sponsors, and owners

"None of the authors have any financial conflict of interests to declare with respect to this study but did contribute to the development of the program, as well as its evaluation."

5-ii) Describe the history/development process

This was detailed in the protocol paper which is referenced in the current manuscript.

5-iii) Revisions and updating

no up-dates

5-iv) Quality assurance methods

Not done

5-v) Ensure replicability by publishing the source code, and/or providing screenshots/screen-capture video, and/or providing flowcharts of the algorithms used

Study images available in protocol paper and 3 month outcome paper

5-vi) Digital preservation

Only preserved via screenshots

5-vii) Access

"After consent, the website generated a personalized link that was sent to the participant's email address. This enabled the participant to create their own study username and password. Next, participants were directed to an online baseline survey. Finally, they were randomized using a fully automated system (one-to-one allocation ratio and with permuted blocks of four). Participants, who were not eligible for the study, were provided with information about other potentially relevant websites and community resources."

"Participants received AU$20 for each baseline and follow-up assessment"

5-viii) Mode of delivery, features/functionalities/components of the intervention and comparator, and the theoretical framework

"We based the intervention on principles from motivational interviewing (MI) and cognitive behavioral therapy (CBT) and adapted from a face-to-face intervention evaluated in amphetamine users [38]. We also adapted the 'decision balance' approach [39] and asked participants to list both the pros and cons of ATS consumption and the potentially good and bad outcomes anticipated from changing their use of ATS. To help participants change their drug use, we assisted them in the development of skills and strategies to aid in behavioral change (e.g. identifying people who could assist them, approaches to help in controlling urges and overcoming cravings, refusal skills, and an action plan to deal with high-risk situations)"

5-ix) Describe use parameters

"The intervention group was provided with immediate access to the first module. We recommended one module be completed per week, but participants were able at advance at their own pace. However, to progress through the program, each page of a module had to be 'opened' in sequence to finish that module and progress to the next. Participants could return to any page or module that had already been accessed."

5-x) Clarify the level of human involvement

None: "fully automated system"

5-xi) Report any prompts/reminders used

"Participants received a reminder email three days after the expected start date if the first module had not commenced, with a further email sent at day seven when the next module was due. This pattern of emails was repeated for the other modules. Participants were sent further emails at three and six months inviting them to complete the follow-up surveys. There was also opportunity at six months to provide feedback on the intervention"

5-xii) Describe any co-interventions (incl. training/support)

None

6a) CONSORT: Completely defined pre-specified primary and secondary outcome measures, including how and when they were assessed

"The study's primary outcome measure was ATS use assessed with the Alcohol, Smoking, Substance Involvement Screening Test (ASSIST) [26]. We also collected secondary outcomes on: (i) help-seeking intentions (General Help-Seeking Questionnaire) [27]; (ii) actual help-seeking (Actual Help-Seeking Questionnaire) [28, 29]; (iii) psychological distress (Kessler 10) [30]; (iv) poly-drug use measured by the ASSIST [26]; (v) quality of life (European Health Interview Survey Quality of Life scale (EUROHIS)) [31]; (vi) days partial or wholly out of role [32]; and (vii) 'readiness to change' (Readiness to Change Questionnaire (RTCQ)) [33]. Demographic details (e.g., age, sex), history of drug use (e.g. age of first use of ATS) and severity of dependence (SDS) [34] were collected as part of the baseline survey. The feedback survey included free text fields plus the 16-item Internet Intervention Adherence Questionnaire [35] and the 16-item Satisfaction with Service measure adapted from the ANU Wellbeing study to reference ATS use rather than depression [36]."

6a-i) Online questionnaires: describe if they were validated for online use and apply CHERRIES items to describe how the questionnaires were designed/deployed

Not validated for online use bar the 16-item Internet Intervention Adherence Questionnaire

6a-ii) Describe whether and how "use" (including intensity of use/dosage) was defined/measured/monitored

"each page of a module had to be 'opened' in sequence to finish that module and progress to the next."
"per protocol' analysis where the 'group' variable was replaced with a variable representing exposure to the intervention ("completed at least one module", "completed no modules" or "control group".)"

6a-iii) Describe whether, how, and when qualitative feedback from participants was obtained

Qualitative feedback was obtained in the development stage as described in the protocol paper. Intervention participants could also provide feedback on the modules

6b) CONSORT: Any changes to trial outcomes after the trial commenced, with reasons

none

7a) CONSORT: How sample size was determined

7a-i) Describe whether and how expected attrition was taken into account when calculating the sample size

"We determined the sample size required to evaluate the primary outcome (ATS score) at a power of 0.8 to detect a medium effect size (e.g. d=0.5) [37]. This required 60 people per group, but allowing for 20% attrition, we recruited a total of 80 people per group"

7b) CONSORT: When applicable, explanation of any interim analyses and stopping guidelines

No stopping guidelines: a 3 month outcome was specified in the protocol

8a) CONSORT: Method used to generate the random allocation sequence

Fully automated "were randomized using a fully automated system (one-to-one allocation ratio and with permuted blocks of four)."

8b) CONSORT: Type of randomisation; details of any restriction (such as blocking and block size)

"were randomized using a fully automated system (one-to-one allocation ratio and with permuted blocks of four)."

9) CONSORT: Mechanism used to implement the random allocation sequence (such as sequentially numbered containers), describing any steps taken to conceal the sequence until interventions were assigned

Fully automated

10) CONSORT: Who generated the random allocation sequence, who enrolled participants, and who assigned participants to interventions

Fully automated

11a) CONSORT: Blinding - If done, who was blinded after assignment to interventions (for example, participants, care providers, those assessing outcomes) and how

11a-i) Specify who was blinded, and who wasn't

the three analysis was conducted blind to allocation

11a-ii) Discuss e.g., whether participants knew which intervention was the "intervention of interest" and which one was the "comparator"

Yes, it was clear if they were waitlist or active group

11b) CONSORT: If relevant, description of the similarity of interventions

n/a

12a) CONSORT: Statistical methods used to compare groups for primary and secondary outcomes

"The primary analysis used an intention-to-treat approach with the critical measure being a time by group interaction. To analyze the correlated data we used multi-level modeling of the repeated measures with generalized estimating equations (GEE). GEE overcomes many of the limitations of traditional repeated measures analysis of variance in that it does not assume homogeneity of correlations over repeated waves and avoids the exclusion of cases with non-complete data [41]. It is also robust to misspecification of the correlation structure [42]. For continuous measures we used an unstructured correlation matrix with a normal distribution and identity link. However, the measures of 'days out of role', intended help-seeking and actual help-seeking were positively skewed, so a Poisson distribution with a log link was used in these analyses. For 'readiness-to-change', we used a multinomial distribution and cumulative logit link. Models were evaluated as the group (intervention, control) by time (baseline, three, six months) interaction"

12a-i) Imputation techniques to deal with attrition / missing values

"We imputed missing data using an iterative Markov chain Monte Carlo (MCMC) method to generate 25 sets of data."

12b) CONSORT: Methods for additional analyses, such as subgroup analyses and adjusted analyses

"We also conducted, a 'per protocol' analysis where the 'group' variable was replaced with a variable representing exposure to the intervention ("completed at least one module", "completed no modules" or "control group".)"

RESULTS

13a) CONSORT: For each group, the numbers of participants who were randomly assigned, received intended treatment, and were analysed for the primary outcome

See consort flow diagram

13b) CONSORT: For each group, losses and exclusions after randomisation, together with reasons

Yes yes flow diagram

13b-i) Attrition diagram

No diagram other than the consort figure.

14a) CONSORT: Dates defining the periods of recruitment and follow-up

"Recruitment ran from January to July 2013"

14a-i) Indicate if critical "secular events" fell into the study period

no

14b) CONSORT: Why the trial ended or was stopped (early)

n/a

15) CONSORT: A table showing baseline demographic and clinical characteristics for each group

Yes see table 1

15-i) Report demographics associated with digital divide issues

Data on education and employment published at 3

16a) CONSORT: For each group, number of participants (denominator) included in each analysis and whether the analysis was by original assigned groups

16-i) Report multiple "denominators" and provide definitions

This is done at a general level - e.g. n who completed follow-up: multiple imputation and per protocol analysis. However, those missing data on a specific variable are not reported.

16-ii) Primary analysis should be intent-to-treat

"The primary analysis used an intention-to-treat approach with the critical measure being a time by group interaction. "

17a) CONSORT: For each primary and secondary outcome, results for each group, and the estimated effect size and its precision (such as 95% confidence interval)

table 2 shows b and standard error. Significant group by time interaction also show odds ratio and 95% CI e.g. "There was a significant group by time interaction for actual help-seeking (b 0.85, p=0.034, rate ratio (RR) = 2.33, 95% CI, 1.07-5.10)"

17a-i) Presentation of process outcomes such as metrics of use and intensity of use

" In terms of 'exposure to the intervention', among the intervention group 30 (37%) people did not start or complete the first module, six (7%) completed one module only, six completed two modules only and 39 (48%) completed all three modules."

17b) CONSORT: For binary outcomes, presentation of both absolute and relative effect sizes is recommended

n/a

18) CONSORT: Results of any other analyses performed, including subgroup analyses and adjusted analyses, distinguishing pre-specified from exploratory

"We also conducted, a 'per protocol' analysis where the 'group' variable was replaced with a variable representing exposure to the intervention ("completed at least one module", "completed no modules" or "control group".)" This was not pre-specified

18-i) Subgroup analysis of comparing only users

"It should be noted that a "per protocol" analysis no longer represents randomised data."

19) CONSORT: All important harms or unintended effects in each group

No harms or unintended effects identified

19-i) Include privacy breaches, technical problems

No harms or unintended effects identified

19-ii) Include qualitative feedback from participants or observations from staff/researchers

"Of the 81 people randomized to the intervention, 35 (43%) provided feedback at six months. Free text responses in particular identified the use of fictional case stories as an engaging approach. The main criticisms included the assumption that people wanted to change their behavior and the lack of information on benefits of drug use (e.g. use of ATS to control symptoms of attention deficit hyperactivity disorder). The most frequently cited negative reactions to the intervention were concerns about privacy (16, 46%) and boredom (7, 20%). Most participants (22, 63%) reported that using the intervention had reduced their adverse drug effects, 86% (30) would recommend the site, 86% endorsed internet delivery, 91% (32) rated the site as easy to use and 91% were satisfied with the program"

DISCUSSION

20) CONSORT: Trial limitations, addressing sources of potential bias, imprecision, multiplicity of analyses

20-i) Typical limitations in ehealth trials

""The representativeness of our sample of ATS users, who were required to have access to the internet, compared with ATS users in general could be questioned. However, at least in Canada, it appears that users of cocaine or cannabis are as likely to have access to the internet as current drinkers [45] and the internet has been shown to be an effective means of reaching hidden populations [46]. Nevertheless, it seems probable that this approach will not reach the most severely disadvantaged ATS users""

21) CONSORT: Generalisability (external validity, applicability) of the trial findings

21-i) Generalizability to other populations

"Firstly, the loss of participants to follow-up threatens the validity and generalizability of conclusions based on these data. However, the rate of attrition (51%) is comparable with the average for fully automated interventions (47%) [44] even though substance users would typically be regarded as a group that is particularly difficult to retain in research projects and treatment"

21-ii) Discuss if there were elements in the RCT that would be different in a routine application setting

Not addressed due to the low impact of the study (e.g. not now intended
for general release at the this stage). However, we do suggest further
work on approach to improve its effectiveness.

22) CONSORT: Interpretation consistent with results, balancing benefits and harms, and considering other relevant evidence

22-i) Restate study questions and summarize the answers suggested by the data, starting with primary outcomes and process outcomes (use)

"The results of the study suggest that this fully automated web-based intervention may be useful to increase help-seeking among people who use ATS in relation to having actually sought help from others. There was also some evidence for a reduction in the number of days completely out of role and increased help-seeking intentions. However, the intervention did not reduce ATS use relative to a waitlist control group. Furthermore, relative to the control group, there was no evidence that the intervention reduced the use of other drugs, improved quality of life or reduced psychological distress"

22-ii) Highlight unanswered new questions, suggest future research

"it may be necessary to further develop aspects of the module that specifically aim to reduce ATS use. There is also the potential to evaluate the intervention as an adjunct to conventional face-to-face treatment. Previous research suggests that compared to a face-to-face CBT intervention alone, online interventions designed to reduce illicit drug use can be effective as an adjunct to weekly individual and group CBT [47]. Furthermore, a web-based program might allow the extent of face-to-face treatment to be reduced and hence lower the burden on service providers and clients inherent in standard treatment."

Other information

23) CONSORT: Registration number and name of trial registry

"Australian and New Zealand Clinical Trials Registry: #12611000947909."

24) CONSORT: Where the full trial protocol can be accessed, if available

Not available

25) CONSORT: Sources of funding and other support (such as supply of drugs), role of funders

"This study was funded by The Commonwealth of Australia, Department of Health and Ageing. Helen Christensen is funded by NHMRC Fellowship 1056964. Kathleen Griffiths is funded by NHMRC Fellowship 1059620. Frances Kay-Lambkin is funded by NHMRC Fellowship 1008972. Robert Tait is funded by a Curtin University Research Fellowship"

X26-i) Comment on ethics committee approval

"he Australian National University Human Research Ethics committee approval the study and it was registered with the Australian and New Zealand Clinical Trials Registry: #12611000947909."

x26-ii) Outline informed consent procedures

"Those who fulfilled the eligibility criteria were invited to provide consent by 'clicking' an onscreen box for each element of the consent form. After consent, the website generated a personalized link that was sent to the participant's email address"

X26-iii) Safety and security procedures

"Participants, who were not eligible for the study, were provided with information about other potentially relevant websites and community resources."

X27-i) State the relation of the study team towards the system being evaluated

"Conflicts of interests
None of the authors have any financial conflict of interests to declare with respect to this study but did contribute to the development of the program, as well as its evaluation.
"
